# Rapid EEG‐Based Detection of Attentional Deficits in Mild Cognitive Impairment

**DOI:** 10.1002/alz70856_105927

**Published:** 2026-01-08

**Authors:** Isaac K. Barss, Robert Trska, Alexandre Henri‐Bhargava, Olav E. Krigolson

**Affiliations:** ^1^ University of Victoria, Victoria, BC, Canada

## Abstract

**Background:**

Mild cognitive impairment (MCI) is a risk factor for dementia, where early detection improves outcomes through early lifestyle change interventions that may delay disease progression. Traditional methods for detecting MCI rely on clinical interviews, cognitive and functional assessments, and laboratory testing, which are resource‐intensive and require skilled administrators. As MCI prevalence grows with an aging population, there is a pressing need for accessible, efficient, and objective screening tools. Electroencephalography (EEG) is sensitive to cognitive dysfunction but is limited by the cost, time, and technical expertise required for traditional systems. Mobile EEG presents a promising alternative, offering quick and user‐friendly assessments. This study focuses on identifying attentional deficits, which are subtle in presentation but impactful on daily living.

**Method:**

We evaluated 200 participants aged 64‐86, including 46 individuals diagnosed with MCI. Cognitive function was assessed using the Repeatable Battery for the Assessment of Neuropsychological Status (RBANS). Participants completed a simple mobile EEG task, the oddball paradigm, designed to elicit the N200 event‐related potential (ERP), which is associated with attentional processes. Multiple linear regression was used to examine the relationship between N200 amplitude and latency and RBANS attention index scores. Welch's t‐test was performed to assess group differences of N200 amplitude between MCI and healthy controls.

**Result:**

N200 amplitude and latency were predictive of RBANS attention index scores (*R* = 0.223). Additionally, MCI patients exhibited reduced N200 amplitude compared to healthy controls (*p* < 0.05).

**Conclusion:**

Our findings support the use of the N200 as a potential electrophysiological biomarker of attentional deficits in MCI. More importantly, they demonstrate the value of mobile EEG for efficient and accessible cognitive screening; our total testing time, including EEG setup, was less than seven minutes and required no technical expertise. This approach could enable scalable early detection of MCI, providing an opportunity for timely intervention in aging populations.

